# Structure-function analysis of *Methanobacterium thermoautotrophicum* RNA ligase – engineering a thermostable ATP independent enzyme

**DOI:** 10.1186/1471-2199-13-24

**Published:** 2012-07-18

**Authors:** Alexander M Zhelkovsky, Larry A McReynolds

**Affiliations:** 1 New England Biolabs, Division of RNA Biology, 240 County Road, Ipswich, MA, 01938-2723, USA

## Abstract

**Background:**

RNA ligases are essential reagents for many methods in molecular biology including NextGen RNA sequencing. To prevent ligation of RNA to itself, ATP independent mutant ligases, defective in self-adenylation, are often used in combination with activated pre-adenylated linkers. It is important that these ligases not have de-adenylation activity, which can result in activation of RNA and formation of background ligation products. An additional useful feature is for the ligase to be active at elevated temperatures. This has the advantage or reducing preferences caused by structures of single-stranded substrates and linkers.

**Results:**

To create an RNA ligase with these desirable properties we performed mutational analysis of the archaeal thermophilic RNA ligase from *Methanobacterium thermoautotrophicum*. We identified amino acids essential for ATP binding and reactivity but dispensable for phosphodiester bond formation with 5’ pre-adenylated donor substrate. The motif V lysine mutant (K246A) showed reduced activity in the first two steps of ligation reaction. The mutant has full ligation activity with pre-adenylated substrates but retained the undesirable activity of deadenylation, which is the reverse of step 2 adenylation. A second mutant, an alanine substitution for the catalytic lysine in motif I (K97A) abolished activity in the first two steps of the ligation reaction, but preserved wild type ligation activity in step 3. The activity of the K97A mutant is similar with either pre-adenylated RNA or single-stranded DNA (ssDNA) as donor substrates but we observed two-fold preference for RNA as an acceptor substrate compared to ssDNA with an identical sequence. In contrast, truncated T4 RNA ligase 2, the commercial enzyme used in these applications, is significantly more active using pre-adenylated RNA as a donor compared to pre-adenylated ssDNA. However, the T4 RNA ligases are ineffective in ligating ssDNA acceptors.

**Conclusions:**

Mutational analysis of the heat stable RNA ligase from *Methanobacterium thermoautotrophicum* resulted in the creation of an ATP independent ligase. The K97A mutant is defective in the first two steps of ligation but retains full activity in ligation of either RNA or ssDNA to a pre-adenylated linker. The ability of the ligase to function at 65°C should reduce the constraints of RNA secondary structure in RNA ligation experiments.

## Background

RNA ligases are widely used as reagents in molecular biology. T4 phage RNA ligases 1 and 2 besides being used for ligation of RNA can be used in rapid amplification of cDNA ends (RLM-RACE) and 3’ RNA labelling [1,2]. T4 RNA ligase 2 is also capable of sealing nicks in dsRNA or dsRNA/DNA hybrids [3]. Phage TS2126 ligase (CircLigase^TM^, ThermoPhage^TM^) is used for ssDNA circularization [4]. The Mth RNA ligase (MthRnl), from thermophilic archaeal bacteria *Methanobacterium thermoautotrophicum*, is used for the enzymatic synthesis of 5’ adenylated DNA linkers [5]. Recently RNA ligases have gained popularity in construction of cDNA libraries for next generation sequencing of small RNAs (for overview, workflow and references see [6,7]).

Capturing small RNAs of unknown sequences is usually achieved by ligation of two linkers with specific sequences on either end of RNA (3’ linker ligation and 5’ linker ligation). This allows specific primers to be used for cDNA synthesis followed by PCR amplification. Modifications of the RNA present challenges for these ligations [8]. For example, some small RNAs are 2’-*O*-methylated at their 3’ ends [9]. The 5’ end of RNA can contain a triphosphate or cap that blocks 5’ ligation unless it is removed [10]. If the RNA contains a 5’-monophosphate and 3’-OH, it can form unwanted by-products, like minicircles or concatamers, which reduce cloning efficiency and require additional enzymatic treatments and purification steps. Alternatively after 3’ linker ligation RNA can be reverse transcribed into first strand cDNA and ligated to a second 3’ linker (5’ ligation independent cloning) [10,11]. Current protocols for capturing small RNAs mostly employ phage T4 RNA ligase 1 (T4Rnl1) and a modified, truncated version of T4 RNA ligase 2 (T4Rnl2tr). However, T4 RNA ligases are either unable to (T4Rnl2) [12] or are slow and inefficient (T4Rnl1) [13,14] in joining two ssDNA molecules relative to joining RNA counterparts. A 5’-phosphorylated or pre-adenylated ssDNA donor substrate may be used, but the acceptor substrate requirements are specific for RNA [15]. In addition, most single-stranded nucleic acids have an increased tendency to fold at low temperature and maintained secondary structure in the presence of Mg^+2^. This is thought to contribute to reducing the efficiency of ligation due to sequestration of hybridized 3’-ends, resulting in ligation bias [7,16]. These structures may lead to a significant reduction in ligation efficiency for certain RNAs. Thermostable phage T2126 ligase might be used in these applications [17,18], but not in the ATP independent ligation.

Overall RNA ligation comprises three linked enzymatic reactions [19]. In the first step, the ligase reacts with ATP to form the intermediate covalent complex with lysine of conserved motif I (step 1). In the second step, AMP from the ligase-adenylate covalent complex is transferred to the 5’-phosphate of an RNA or ssDNA donor substrate, creating an activated 5’ adenylated intermediate product, designated AppRNA or single-stranded AppDNA (step 2). In the third step of a ligation reaction, the phosphodiester bond is formed between the 3’-OH of the acceptor substrate and the 5’ adenylated phosphate of a donor, releasing AMP (step 3). It is difficult to optimize all three biochemical reactions for efficient ligation, due to the different reaction requirements. For example, ATP is strictly required for RNA ligase adenylation, but often inhibits the step 3 of the ligation.

RNA ligation *in vitro*, however, can be performed in two stages. The 5’ pre-adenylated activated donor, the product of the second ligation step, can be synthesized by enzymatic or chemical methods. In a second stage, equivalent to ligation step 3 above, this activated donor substrate is ligated to an acceptor in the absence of ATP. This “split” ligation method was originally described for 3’-linker ligation using T4Rnl1 [20]. An improved method used the T4Rnl2 truncated enzyme, and its derivatives, which have reduced activity in the substrate deadenylation reaction catalyzed by T4Rnl1 [6,21-23]. Ligation of pre-adenylated DNA to RNA in a 3’ linker ligation reaction lacking ATP is also now a preferred approach to ligation of microRNA for next generation sequencing. Since only the 3’ end of the oligonucleotides can be a substrate for this stage, this approach eliminates (i) the need for a dephosphorylation step (removing the 5’-phosphate from small RNAs prior to 3’ linker ligation) to avoid RNA circularization and concatemerization, (ii) thus there is no need for re-phosphorylation for the second ligation (of the 5’ linker) and (iii) eliminates additional purification steps, which cause significant loss of material. Splitting these reactions into two parts increases the efficiency of ligation by enabling optimization of a single step of the ligation reaction and eliminates ATP, which besides producing by-products, if in excess, also can inhibit ligation.

There is a two-fold approach to make the “split” ligation method successful. First, there is a need for a convenient method for synthesis of pre-adenylated linkers. Second, this type of ligation requires non-adenylated ligase (apoenzyme), which can use the pre-adenylated donor as a substrate. The ligase should also be inactive in the reverse step 2 of the ligation reaction, which transfers AMP from activated pre-adenylated substrate back to the enzyme. The pre-adenylated substrates are commercially available or can be efficiently synthesized as described in the Methods [5]. The choice of suitable RNA ligases is limited to the commercially available C-terminal truncated version of T4Rnl2 and its K227Q mutant. This C-terminal deleted version of T4Rnl2 has greatly reduced, but is not completely defective in self-adenylation activity due to pH-optimum shift of the reaction step 1 to alkaline pH [24]. For the same reason truncated T4Rnl2 also retains some deadenylation activity in ligation reaction using a pre-adenylated donor. An additional mutation in motif V was later introduced (K227Q) to increase the fidelity of truncated T4Rnl2 in ligation [6,21] but resulted in reduced ligation activity.

Taken together, there is a need for single-stranded nucleic acid ligase, which could work efficiently at elevated temperature, reduce structural constraints, and ligate ssDNA and 2’-*O*-methylated RNA as well as RNA. In order to eliminate side reactions the ligase should accept pre-adenylated substrates in an ATP independent ligation reaction and not have deadenylation activity. To our knowledge no such ligase exists in nature. Thermostable archaeal RNA ligases are attractive choices for enzyme engineering. Their optimum reaction temperature is 60–65°C and they readily accept ssDNA in circularization reactions, indicating acceptance of deoxy-substrates as a donor as well as an acceptor [4,25-27].

Archaeal MthRnl is a homodimeric, ATP-dependent, thermostable enzyme with optimum activity at 65°C. At this temperature short hybrids should be significantly reduced. MthRnl can carry out intramolecular ligation of either ssDNA or RNA. The circularization of ssDNA is only half as efficient as the circularization of RNA [25]. However, wild type MthRnl has a few shortcomings. While ATP is required for activity, the third step in ligation is inhibited at ATP concentrations as low as 50 μM. It is not suitable for “split” ligation because MthRnl is purified mostly in a stable self-adenylated form, a form unable to carry out the step 3 ligation with pre-adenylated linkers. Recovering deadenylated apoenzyme is inefficient and unpredictable. The apoenzyme also deadenylates pre-adenylated donor, in ligation reactions without ATP.

To overcome these limitations in RNA ligation, we performed a mutagenesis of essential amino acids in the active site of MthRnl to identify and analyze active mutants defective in ATP binding or ATP reactivity, and consequently unable to self-adenylate. These mutants were then characterized for use in the ligation reactions without ATP. Here we present structure-function analysis of these mutants and their activities in single-stranded nucleic acid (RNA and DNA) ligation. Comparison of active MthRnl mutants to T4 RNA ligases revealed significant differences in substrate specificity, which allowed us to define new conditions for single-stranded nucleic acid ligation.

## Results and discussion

### Screening for MthRnl mutants defective in self-adenylation

**Figure 1:**
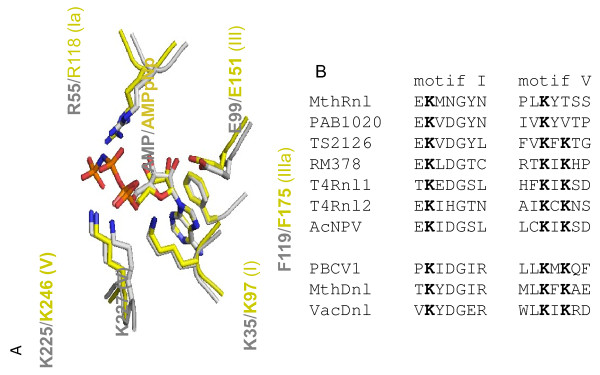
**(A) Structural comparison of T4 RNA ligase 2 and archaeal RNA ligase PAB1020 active sites.** Two structures were superimposed based on coordinates of pre-bound AMP and ATP homolog (AMPPNP) as well as known conserved amino acids of the ligase active sites. The T4Rnl2 structure is represented in grey and PAB1020 in yellow. The numbers of amino acids in the conserved motifs I-V (in parenthesis) are for T4Rnl2 and PAB1020 ortholog MthRnl, which were determined after sequence alignment of two archaeal enzymes. **(B)** The sequences of the conserved motifs I and V of MthRnl compared to corresponding motifs in the RNA and DNA ligases as discussed in the text. The listed RNA ligases are from: MthRnl (*Methanobacterium thermoautotrophicum*), PAB1020 (*Pyrococcus abyssi*), TS2126 (bacteriophage *Thermus scotoductus*), RM378 (bacteriophage *Rhodothermus marinus*), T4Rnl1 and T4Rnl2 (bacteriophage T4), AcNPV (*Autographa californica* nucleopolyhedrovirus). And DNA ligases are from: PBCV1 (*Chlorella* virus), MthDnl (*Methanobacterium thermoautotrophicum*), VacDnl (Vaccinia virus). The conserved lysines are shown in bold.

To guide the choice of amino acids for point mutations we used the structure-function analysis of T4 RNA ligases [24,28-30] and the crystal structure of the closest ortholog of MthRnl, PAB1020 RNA ligase [26]. MthRnl has little sequence homology to characterized RNA ligases. To determine which amino acids of MthRnl belong to conserved motifs I-V of the adenylyltransferase domain we first manually superimposed two known RNA ligase structures, T4Rnl2 (PDB 2HVR) with known functional residues and MthRnl ortholog PAB1020 (PDB 2VUG) using Swiss PDB Viewer (http://www.expasy.org/spdbv/). Then we aligned the sequences of MthRnl with PAB1020. The resulting analysis is shown in Figure 1 (panel A) with numbered amino acids corresponding to the amino acids of T4Rnl2 and MthRnl. The corresponding conserved motifs are indicated within parentheses. Based on published results of mutagenic analysis of RNA and DNA ligases, which showed defects in ATP binding or reactivity, we chose conserved residues of motifs I (K97) and V (K246) for mutagenesis, as well as a short truncation of the C-terminus of MthRnl to generate candidate mutants to screen for the defects in step 1 of the ligation reaction. 

The mutants K97A, K246A, K246Q, the double mutant KK97-246AA and the 17 amino-acid C-terminal deletion were first analyzed for their overall activity over a wide range of pH (5.5–9.5) and ATP concentrations (0–500 μM). The adenylation status of mutants after expression and purification from *E.coli* was analyzed using mass-spectrometry. Non-adenylated apoenzymes defective in overall activity were further analyzed for their activity in isolated steps 1–3 of ligation reaction. This approach resulted in the identification of the ligase mutants defective in steps 1 and 2, but active in step 3 of ligation.

### Analysis of MthRnl C-terminal truncation

Some ATP dependent DNA ligases have motif VI at their C-terminus. This motif is required for formation of the adenylated enzyme, but is dispensable for step 3 ligation with a pre-adenylated nick [31,32]. Deletion of as few as 15 amino acids from the C-terminus of T4Rnl2 produced an enzyme as defective in the step 1 ligation as T4Rnl2tr (also known as T4Rnl2(1-249)) lacking the entire 85 amino acid C-terminal domain [24]. In the PAB1020 ligase, the C-terminus of one subunit of the homodimer is in a position to coordinate a Mg^+2^ ion and the gamma phosphate of ATP bound to the other subunit [26]. Similarly, the complete C-terminal domain of MthRnl is responsible for dimerization, which is required for enzyme activity [25]. We speculated, based on the sequence of MthRnl and the structure of PAB1020, that the last 17 amino acids of MthRnl may play a similar role in interacting with ATP, without interrupting dimerization.

In contrast to this speculation, we found that a 17 amino acid C-terminal truncated version of MthRnl, purified from *E.coli*, is adenylated. The enzyme is “temperature sensitive” losing activity above 50°C as compared to 75°C for wild type MthRnl. At the permissive temperature, below 50°C, the truncated mutant can transfer AMP from the enzyme to a 5’-phosphorylated donor substrate, but is inactive in the overall ligation reaction. These results demonstrate that C-terminal deletion of MthRnl is not a useful path for disrupting ATP binding to obtain active apoenzyme for “split” ligation.

### Analysis of MthRnl motif V mutants

Motif V studies of different RNA and DNA ligases demonstrate the role of this motif in ATP binding and the first step of the reaction, enzyme adenylation. Motif V of MthRnl is slightly different from the typical motif found in most RNA and DNA ligases, where two lysines (KxK) are essential for the overall ligation reaction. In MthRnl and some other archaeal RNA ligases, a non-nucleophilic amino acid (threonine, T248 in MthRnl) is substituted for the second lysine in this motif (Figure 1B). In PAB1020 RNA ligase the corresponding position contains valine (hidden for clarity in Figure 1A). As shown in Figure 1A, K246 of MthRnl is the first lysine in the motif, corresponding to K225 of T4Rnl2. K225A and K225Q mutants of motif V of T4Rnl2 are inactive in self-adenylation [29].

**Figure 2:**
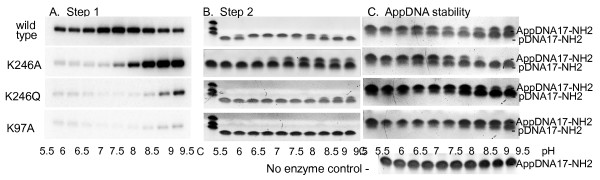
**Activity of the MthRnl and the K246A, K246Q and K97A mutants in different steps of the ligation reaction.(A)** pH-dependence of self-adenylation activity of enzymes (step 1) in reactions with radioactive ATP. The reactions were carried out as described in the Methods and the products were resolved by an SDS-PAGE. Autoradiographs of the gels are shown. **(B)** pH-dependence of adenylation of 5’-phosphorylated, 3’-amino-blocked ssDNA (pDNA17-NH_2_) (step 2) with wild type and the mutants of MthRnl under reaction conditions described in the Methods. The reaction products were separated by 15% urea-PAGE and visualized with SYBR Gold stain. **(C)** pH-dependence of deadenylation/hydrolysis of 5’ pre-adenylated DNA (AppDNA17-NH_2_) with wild type and the mutants of MthRnl under reaction conditions described in the Methods. The products were analyzed on 15% urea-PAGE and stained with SYBR Gold. Enzymes used in each step of ligation reactions are indicated on the left. Position of DNA donor substrates and products are indicated on the right. The pH of the buffer is indicated at the bottom. The control (c) indicates the reaction without enzyme.

Motif V mutants K246A and K246Q of MthRnl were purified from *E.coli* as non-adenylated apoenzymes as determined by MS-TOF analysis. Further analysis revealed that the pH-optimum of step 1 reaction self-adenylation was shifted from 7.0–7.5 for the wild type enzyme to 9.0–9.5 for the K246A mutant (Figure 2, panel A). The Km for ATP binding for this step of the ligation reaction increased from 0.9 μM for wild type to 60 μM for K246A at their corresponding pH-optima. The pH shift was even more severe for the K246Q mutant (Figure 2, panel A). Thus, the K246 mutations did not preclude the reaction of MthRnl with ATP, but changed ATP binding and the pH-optimum of the reaction, which may explain the defect in self-adenylation when produced in *E.coli*.

**Figure 3:**
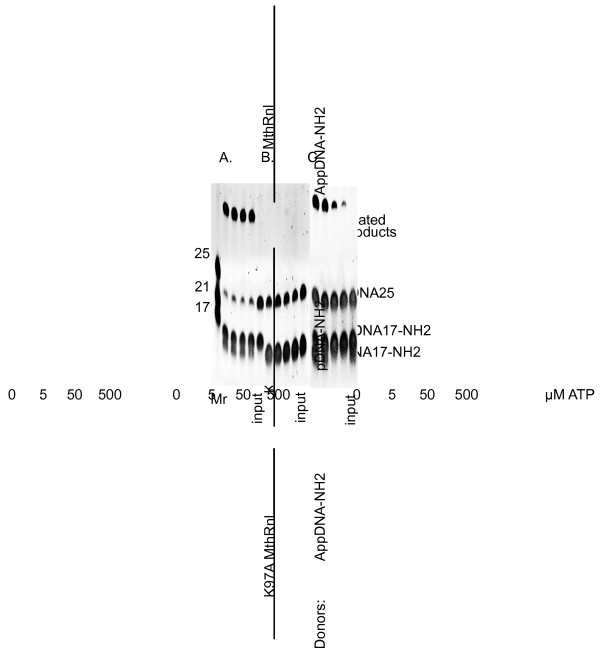
**ATP independence of the K97A MthRnl mutant.(A)** The reactions of 5’ pre-adenylated 3’-amino-blocked DNA donor (AppDNA17-NH_2_) with ssDNA acceptor (DNA25) and K97A MthRnl mutant without ATP or with increasing (5–500 μM) concentration of ATP. **(B)** The reaction of 5’-phosphorylated 3’-amino-blocked DNA donor (pDNA17-NH_2_) with ssDNA acceptor (DNA25) and K97A MthRnl mutant without ATP or with increasing (5–500 μM) concentration of ATP. **(C)** The reactions of 5’ pre-adenylated 3’-amino blocked DNA donor (AppDNA17-NH_2_) with ssDNA acceptor (DNA25) and wild type MthRnl without ATP or with increasing (5–500 μM) concentration of ATP. The ligation reactions were carried out as described in the Methods. The products were separated on 15% urea-PAGE and visualized with SYBR Gold stain. The concentration of ATP used in reactions is indicated at the bottom. Positions of substrates and ligated products are indicated on the right. Mr are molecular weight RNA markers. The ‘input’ indicates reactions without enzyme.

The K246A mutant under the assay conditions described in the Methods shows reduced activity in step 2 in comparison to wild type enzyme, with the pH optimum of the reaction shifted to 8.0–8.5 (Figure 2, panel B). However, when the ATP concentration is increased to 500 μM, and the pH is adjusted to 8.5, the K246A mutant is only slightly less active than the wild type enzyme in the step 2 and overall ligation compared at their optimal conditions. These properties make K246A mutant more efficient in donor substrate labeling by incorporating radioactive AMP to form a 5’-adenylated linker [21] in comparison to wild type MthRnl, which is purified pre-adenylated with non-radioactive AMP and overall is a low turnover enzyme. In wild type MthRnl, ATP is a strong inhibitor of overall ligation leading to the release and accumulation of intermediate products, pre-adenylated donor substrates [5,25] (see also Figure 3, panel C). In contrast, an elevated ATP concentration does not inhibit the overall ligation, as measured in a DNA circularization reaction mediated by the K246A mutant. The activity of the K246Q mutant is greatly compromised in step 2 of the ligation reaction. The activity optimum is shifted to alkaline pH, similar to the self-adenylation reaction (Figure 2, panel B) with only trace activity in overall ligation at pH 8.5. However, both mutants are active in the isolated step 3 of the ligation reaction with pre-adenylated substrate without ATP (see below). 

### Analysis of MthRnl motif I mutant

The lysine in motif I is a catalytic residue in RNA and DNA ligases and the site for enzyme-AMP covalent complex formation. Mutation of this lysine absolutely abolished ligase self-adenylation and subsequent transfer of AMP to the 5’-phosphate of the donor substrate. In contrast, the effect of mutation is somewhat variable among ligases for step 3 of the ligation reaction when pre-adenylated substrate is used without ATP. Mutation of the corresponding lysine (K99) in T4Rnl1 to alanine inactivates the enzyme in all three steps of the ligation reaction, as was shown in reactions with a 5’-phosphorylated oligo and ATP as well as with pre-adenylated substrate without ATP [30]. For baculovirus AcNPV Rnl1, the K103A mutant displays 6% of wild type activity in the step 3 reaction [33]. For T4Rnl2, the K35A mutant is partially active with a pre-adenylated substrate without ATP in step 3 ligation [29]. The fact that the catalytic lysine is not strictly essential for phosphodiester bond formation for ATP-dependent DNA ligases was reported for Chlorella virus DNA ligase (K27A mutant) [31], Mth DNA ligase (K251A mutant) [34] and vaccinia virus DNA ligase (K231A) [35].

As shown in Figure 1, the predicted catalytic lysine of motif I in MthRnl is K97. The alanine substitution (K97A) produced, as expected, an enzyme inactive in steps 1 and 2 (Figure 2, panels A and B). As expected it was also inactive in the overall ligation reaction with a 5’-phosphorylated substrate and ATP, and subsequent donor substrate adenylation (Figure 3, panel B). However, as with DNA ligases, this mutant is active in the isolated step 3 of the ligation reaction with a pre-adenylated substrate (Figure 3, panel A). The K97A mutation made the enzyme insensitive to ATP (Figure 3, panel A) in comparison to the wild type enzyme (Figure 3, panel C) in the reactions with the pre-adenylated donor.

Interestingly, the double mutation of the motifs I and V amino acids K97A and K246A in MthRnl produced totally inactive enzyme in all ligation steps, indicating some interplay between these two lysines.

### Analysis of MthRnl mutants in step 3 of the ligation reaction with pre-adenylated substrate

**Figure 4:**
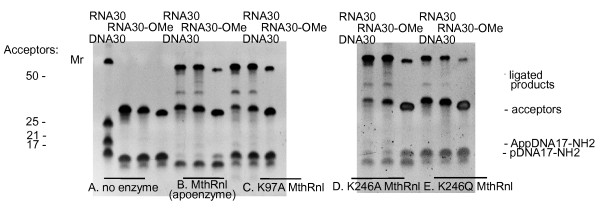
**A comparison of the wild type MthRnl and the MthRnl mutants in step 3 of the ligation reaction with various acceptors.** Enzymes were assayed using three different acceptors with identical sequence: RNA (RNA30), 2’-*O*-methylated at 3’-end RNA (RNA30-OMe) or ssDNA (DNA30), and the donor AppDNA17-NH_2_ as described in the Methods. **(A)** The control reactions without enzyme. **(B)** The reactions with non-adenylated form of the wild type MthRnl. **(C, D and E)** The reactions with K97A, K246A and K246Q MthRnl mutants respectively. The products were separated on 15% urea-PAGE and visualized with SYBR Gold stain. Positions of substrates, ligated products and by-product pDNA17-NH_2_ are indicated on the right. Acceptor substrates used in the reactions are indicated on the top. Mr indicates molecular weight RNA markers.

The wild type MthRnl apoenzyme, and the self-adenylation defective mutants K97A, K246A and K246Q, were further analyzed for their activity in step 3 of the ligation reaction with the pre-adenylated donor without ATP (Figure 4). It is a challenging but more practical assay than intramolecular circularization employed in the literature. It allowed testing different RNA, ssDNA and their combination as acceptors or pre-adenylated donors. In this assay, three acceptor substrates of identical sequence were tested: RNA oligonucleotide (RNA30), RNA with 2’-*O*-methylated 3’-end (RNA30-OMe), and ssDNA oligonucleotide (DNA30). As shown, all tested enzymes were active in step 3 of the ligation reaction with pre-adenylated donor without ATP. The least active mutant was K246Q (Figure 4, panel E). The K97A and K246A mutants have ligation activity equal to or better than wild type enzyme (Figure 4, panels C and D). Non-adenylated wild type enzyme and all tested mutants were able to ligate RNA with modified 3’-end only slightly less efficiently than RNA. The ligation of ssDNA to pre-adenylated DNA was about half as efficient as the ligation of RNA to pre-adenylated DNA using the K97A mutant (Figures 4, panel C and 5, panels C and F).

**Figure 5:**
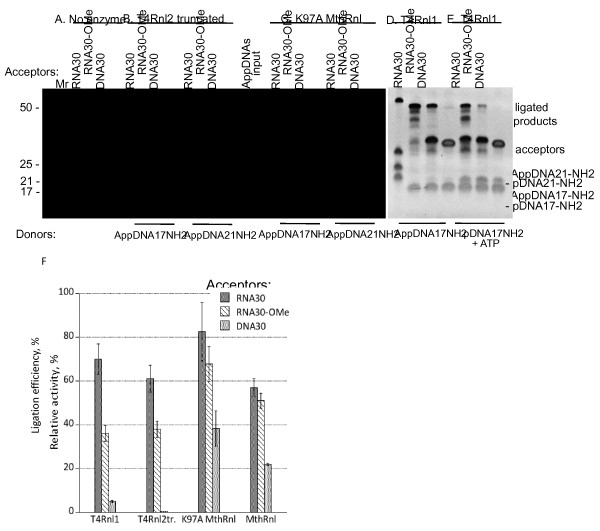
**A comparison of the K97A MthRnl mutant activity with activities of truncated T4Rnl2 and T4Rnl1 in step 3 of the ligation reaction.** Enzymes were assayed using three different acceptors with identical sequence: RNA (RNA30), 2’-*O*-methylated at 3’-end RNA (RNA-OMe) or ssDNA (DNA30) in reactions with 5’ pre-adenylated DNA donors AppDNA17-NH_2_ or AppDNA21-NH_2_ as described in the Methods. **(A)** Control reactions without enzyme. **(B, C and D)** Reactions with truncated T4Rnl2, K97A MthRnl mutant and T4Rnl1 respectively. **(E)** For comparison, T4Rnl1 was assayed in the ligation reactions with 5’-phosphorylated 3’-amino-blocked DNA donor (pDNA17-NH_2_) and indicated acceptors in presence of ATP. (F) Percentage of ligation of the different acceptors was calculated from experiments shown in Figures 4 (panels **B** and **C**) and 5 (panels **B**, **C** and **D**). Mean values and standard deviation were calculated from multiple (2–5) gels analysis described in the Methods are shown.

The reversibility of the step 2 of the ligation reaction was implicated in the circularization of 5’-phosphorylated acceptor substrates in the ligation reactions without ATP. This occurs when pre-adenylated donor substrate reactivates ligase by transferring 5’-AMP back to the catalytic lysine of the enzyme. This allows the adenylation and intramolecular ligation of the 5’-phosphorylated acceptor. As shown in Figure 2 (panel C), in an assay to test the stability of the pre-adenylated substrate with different mutants, within the pH range of ligation reaction (6.5–7.5), the K97A and K246Q mutants did not efficiently deadenylate/hydrolyze the pre-adenylated substrate. Under the same conditions the wild type enzyme and the K246A mutant did deadenylate the pre-adenylated substrate. This effect is even more evident in step 3 of the ligation reactions, where the acceptor substrate is also present (Figure 4, panels B and D).

Taken together, the K97A mutant is our best choice for ligation of single-stranded nucleic acids in the applications requiring lack of ATP.

### Comparison of K97A MthRnl to T4 RNA ligases

The ligation efficiency of K97A MthRnl in “split” ligation was compared to T4Rnl1 and truncated T4Rnl2 using various substrates (Figure 5). While the higher ligation rates could be achieved with T4 RNA ligases by extending the reaction time, lowering temperature and adding polyethylene glycol [8], all the same could be achieved with the K97A mutant. For comparison we kept the reaction conditions as similar as possible as described in the Methods.

The K97A MthRnl and truncated T4Rnl2 have similar properties: both enzymes are defective in first two steps of ligation reaction and both require pre-adenylated donor substrate. Although direct comparison has limitations because of different reaction conditions, the major differences are evident. First, K97A MthRnl is more efficient for ssDNA ligation. Figure 5 (panel F) shows a quantitative comparison of ligation of three acceptors (RNA, 2’-*O*-methylated-RNA and ssDNA) containing identical sequence with pre-adenylated donor (AppDNA17-NH_2_), using four enzymes: MthRnl apoenzyme, K97A MthRnl, truncated T4Rnl2 and T4Rnl1. While RNA acceptor ligations show similar efficiency, T4Rnl2tr discriminates strongly against DNA (Figure 5, panel B and F).

The current protocol for ssDNA ligation employs T4Rnl1 [10]. When this enzyme was tested with pre-adenylated donor, RNA ligation was comparable with K97A MthRnl. But when ssDNA was ligated to the adenylated linker less than 5% of the substrate was converted to product (Figure 5, panel D and F). In addition, T4Rnl1 can efficiently de-adenylate pre-adenylated substrates. In reaction with 5’-phosphorylated donor, substrate and ATP, the ligation with T4Rnl1 ligase is even less efficient in identical conditions than with pre-adenylated substrate (Figure 5, panel E).

The T4 RNA ligases accept 2’-*O*-methylated substrates, albeit ligation is less efficient compared to unmodified RNA [8], (Figure 5, panels B, D, E and F). The K97A mutant is less discriminatory with 2’-*O*-methylated 3’-end substrates (Figures 4, panel C and 5, panels C and F).

The ligation efficiency depends at least partially on folding or hybridization of single-stranded nucleic acid substrates in the reaction [7,36,37]. As a proof of concept, shown in Figure 5, ligation using the K97A mutant at 65°C is less sensitive to substitution of one pre-adenylated donor substrate (AppDNA17-NH_2_) for another (AppDNA21-NH_2_), in comparison to truncated T4Rnl2 at 25°C (Figure 5, panels B and C). Understanding potential preferences for RNA sequences or structures will require further study.

**Figure 6:**
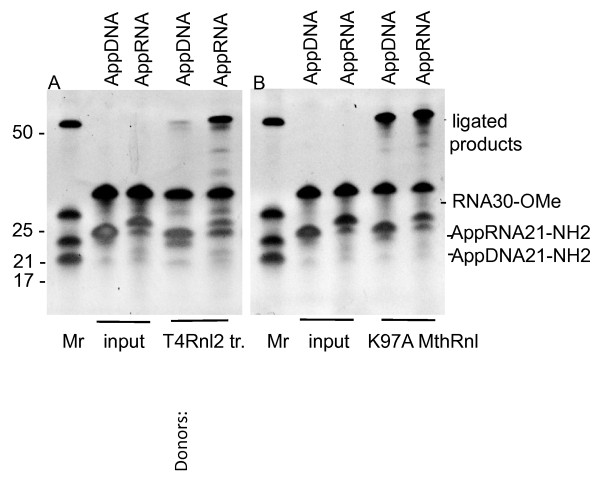
**The comparison of pre-adenylated RNA or DNA donors in step 3 of the ligation reaction with the K97A MthRnl mutant or truncated T4Rnl2.** Enzymes were assayed using 2’-*O*-methylated at 3’-end RNA (RNA-OMe) acceptor in reactions with 5’ pre-adenylated RNA (AppRNA21-NH_2_) or DNA (AppDNA21-NH_2_) with identical sequence. **(A)** Reactions without (input) or with truncated T4Rnl2. **(B)** Reactions without (input) or with K97A MthRnl mutant. Reactions were carried out as described in the Methods at 25°C for T4Rnl2tr and 65°C for K97A mutant. The products were analysed on 15% urea-PAGE and stained with SYBR Gold stain. Positions of substrates and ligated products are indicated on the right. Donor substrates used in the reactions are indicated on the top. Mr indicates molecular weight RNA markers.

Non-adenylated RNA ligases in general can accept either RNA or DNA adenylated donor substrates [15]. This lack of discrimination between RNA or DNA is widely used in 3’ ligation protocols where pre-adenylated ssDNA linkers are ligated to RNA. Pre-adenylated DNA linkers are more commercially accessible substrates than pre-adenylated RNAs. To test donor substrate specificity of the K97A MthRnl we prepared pre-adenylated RNA linker (AppRNA21NH_2_) with sequence identical to pre-adenylated DNA (AppDNA21NH_2_) using the protocol described for 5’DNA adenylation [5]. Then we assayed both substrates side-by-side in the ligation reactions with the least efficient tested acceptor (Figure 5), an RNA30-OMe. In addition we compared the specificity of truncated T4Rnl2 to K97A MthRnl with the same substrates. As shown in Figure 6, truncated T4Rnl2 is significantly more active when both substrates are RNA (Figure 6, panel A). K97A MthRnl ligates either pre-adenylated DNA or RNA donors with similar efficiency (Figure 6, panel B). This might have been anticipated, knowing ssDNA circularization activities of archaeal RNA ligases [4,25]. From a practical point of view, based on popularity of ATP independent ligation in RNA research, the increased ligation efficiency of truncated T4 Rnl2 when using AppRNA donors, compared to AppDNA should be explored. 

The precise function of archaeal RNA ligases is not known. It is tempting to speculate that they are involved in RNA splicing/repair but with a somewhat different mechanism from the T4 RNA ligases. While these two classes of RNA ligases have similarities, the striking difference between the two is their ability to ligate single-stranded DNA. There may be the possibility that the Mth RNA ligase also has a role in DNA ligation.

## Conclusions

Using structural information from related RNA ligases we made targeted point mutations and truncations to create an ATP independent archaeal ligase. The objective was to create an enzyme that was defective in either self-adenyadenylation or adenylation of the donor, steps 1 and 2, but retained full ligation activity, step 3 in the reaction. We identified a mutation, K97A, which met these criteria. Because this mutant of Mth RNA ligase is ATP independent, it requires an adenylated RNA or DNA linker for ligation. This is a useful property that reduces background ligation of RNA to itself and improves the efficiency of library construction. The enzyme can ligate either single stranded RNA or DNA to an adenylated linker at 65°C. These properties of thermostability and the ability to use either RNA or ssDNA as acceptors in a ligation reaction make this enzyme useful for multiple applications in molecular biology.

## Methods

### Enzymes

The MthRnl open reading frame was codon optimized for expression in *E.coli*[38], synthesized by DNA2.0 and subcloned into the Nde I/BamH I sites of pET16b plasmid (Novagen) in frame with a His_10_ N-terminal tag as described previously [25]. This construct was used as a template to produce MthRnl with single amino acid substitutions using a Stratagene Quick Change II site-directed mutagenesis protocol (Agilent Technologies). A 17 amino-acid C-terminal truncation was constructed by PCR amplification, which introduced a new stop codon. The resulting constructs were sequenced and expressed in T7 Express *lysY/I*^*q*^*E.coli* cells (NEB) as follows.

After induction with 0.5 mM IPTG, 2 liters of cell cultures with OD^600^ 0.5 were shaken for an additional three hours at 30°C, harvested and resuspended in 50 mM Tris-HCl pH 7.5 buffer, 1 M NaCl, 10% glycerol. After sonication and clearing by centrifugation, extracts were loaded on a 5 ml Ni-NTA Superflow column (Qiagen) and washed with 10 column volumes (cv) of resuspension buffer supplemented with 10 mM imidazole. The column was washed with 5 cv of 10 mM Tris-HCl pH 7.5 buffer, 50 mM NaCl, 10% glycerol and 100 mM imidazole and then bound proteins were eluted with 5 cv of the same buffer containing 300 mM imidazole. The eluted proteins were diluted two-fold with 10 mM Tris-HCl pH 7.5 buffer, 10% glycerol, 1 mM EDTA, 1 mM DTT (dilution buffer) and loaded on a 5 ml HiTrap Heparin column (GE Healthcare). After washing with 5 cv of dilution buffer with 50 mM NaCl, the protein was eluted with 20 cv of a 0.05–1.0 M NaCl gradient in the same buffer. Fractions containing expressed MthRnl or MthRnl mutants (400–600 mM NaCl) were combined and dialyzed overnight in dilution buffer supplemented with 50 mM NaCl and 50% glycerol, and stored at −20°C. Protein concentration was determined using a Bradford assay (BioRad) with BSA as a standard. A typical yield of purified proteins was 5–7 mg from a one-liter cell culture.

The state of enzyme adenylation was analyzed by mass-spectrometry using ESI-TOF 6210 (Agilent Technologies) and by testing for DNA adenylation in the absence of ATP [5]. Wild type MthRnl as purified from *E.coli* is mostly pre-adenylated, with an occasional batch mostly non-adenylated. This non-adenylated apoenzyme was used in this study to compare activities with MthRnl mutants.

T4 RNA ligases 1 (T4Rnl1) and T4 RNA ligase 2 truncated (T4Rnl2tr) were from NEB.

### Substrates

Oligonucleotides used in ligation assays were synthesized at Integrated DNA Technologies. Preparative adenylation of DNA and RNA linkers (donor substrates) were performed using 5’-DNA Adenylation kit (NEB) [5] Table 1. 

**Table 1:** Substrates

**Acceptor substrates:**	
UCC UAU GAU GCA GGC CUU ACU AGG UGC AGU	(RNA30)
UCC UAU GAU GCA GGC CUU ACU AGG UGC AGU-OCH_3_	(RNA30-OMe)
TCC TAT GAT GCA GGC CTT ACT AGG TGC AGT	(DNA30)
CAT TGT AGC CGT CCA TCT TTT CCT C	(DNA25)
**Donor substrates:**	
pCTG TAG GCA CCA TCA AT-NH_2_	(pDNA17-NH_2_)
AppCTG TAG GCA CCA TCA AT-NH_2_	(AppDNA17-NH_2_)
AppTCG TAT GCC GTC TTC TGC TTG-NH_2_	(AppDNA21-NH_2_)
AppUCG UAU GCC GUC UUC UGC UUG-NH_2_	(AppRNA21-NH_2_)

### Reaction conditions

Overall ligation activity of MthRnl and its mutants was assayed as described previously, using an ssDNA circularization assay [5]. All reactions, except self-adenylation, were carried out in single-turnover conditions, where concentrations of enzymes were close to equimolar or in excess of the substrates (the active form of MthRnl is a homodimer).

Self-adenylation of the enzymes were performed in 10 μl of a reaction mixture containing 10 pmol of MthRnl or MthRnl mutant (460 ng), 200 μM ATP and 1 μCi of α-[^32^P]-ATP (Perkin-Elmer), 10 mM Mg^+2^ for 30 min at 65°C in 10 mM bis-Tris propane-HCl buffer with corresponding pH@ 25°C indicated in the figures. For ATP binding assay, the same reactions were performed at pH 7.0 and 8.5 for the wild type MthRnl and the K246A mutant respectively with variable concentration (0–200 μM) of ATP. Reaction products were separated on 10–20% Tris-Glycine SDS polyacrylamide minigels (Invitrogen), visualized with Simply Blue Safe Stain (Invitrogen) and after gel drying exposed to a PhosphoStorage Screen (Bio-Rad) and scanned on a Typhoon 9400 Imager (GE Healthcare). Visualized radioactive bands were quantitated using Image Quant TL software (GE Healthcare). Alternatively, protein bands were isolated and radioactivity counted with a Liquid Scintillation Analyzer 2100TR (Packard).

The ssDNA adenylation assay was performed in 10 μl of reaction mixture containing 10 mM bis-Tris propane-HCl buffer, pH 6.5, 10 mM Mg^+2^, 5 pmol 5’-phosphorylated 3’ blocked DNA, 10 pmol of MthRnl or MthRnl mutant (460 ng), 100 μM ATP for 60 min at 65°C in 200 μl PCR tubes using an S1000 Thermal Cycler (BioRad), followed by inactivation of the enzyme at 90°C for 3 min. After the addition of 5 μl formamide loading buffer, the reaction mixture was separated on 15% Urea–TBE denaturing polyacrylamide minigels (Invitrogen), stained with SYBR Gold (Invitrogen) and visualized using AlphaImager HP (Alpha Innotech). All parameters that are different from the standard reaction conditions are indicated in the figure legends.

The deadenylation/hydrolysis reactions were performed under conditions described for the ssDNA adenylation reaction, except that pre-adenylated DNA was substituted for 5’ phosphorylated DNA, and ATP was omitted from the reaction.

The ligation of pre-adenylated ssDNA or RNA donors with a ssDNA or RNA acceptor without ATP was performed in a reaction mixture (10 μl total) containing 10 mM bisTris-Propane buffer, pH 7.0 (@25°C), 10 mM Mg^+2^ for RNA ligation or 5 mM Mn^+2^ for ssDNA ligation, 5 mM DTT, 5 pmol of acceptor substrate, 8 pmol of pre-adenylated donor substrate, 20 pmol (monomer) MthRnl or MthRnl mutant. Assays were performed as described for ssDNA adenylation reaction. For quantitation, gel images were analyzed using Quantity One software (Bio-Rad). All parameters that are different from the standard reaction conditions are indicated in the figure legends.

The ligation of pre-adenylated ssDNA or RNA with an acceptor substrate using T4 RNA ligase 1 or truncated T4 RNA ligase 2 (NEB) without ATP were carried out in single-turnover conditions and performed in 10 μl containing 5 pmol of the RNA acceptor, 8 pmol of pre-adenylated donor substrate in 10 mM Tris-HCl pH 7.5 buffer, 10 mM Mg^+2^, 1 mM DTT and 200 U (~10 pmol) of truncated T4Rnl2 or 10 U (~50 pmol) of T4Rnl1. Reactions were incubated for 2 hours at 25°C. Standard reactions with a 5’-phosphorylated donor, various acceptors and ATP using T4Rnl1 were performed according to the manufacturer’s protocol (NEB). Reactions were stopped by adding 5 μl formamide loading buffer, heat inactivated at 90°C for 3 min and the products were separated, stained and visualized as described for the ssDNA adenylation above.

## Competing interests

LM and AZ are employees of New England Biolabs, a company that sells mutant Mth RNA ligase and other proteins for RNA and DNA research.

## Authors’ contributions

AZ designed, carried out experiments and drafted the manuscript. LM discussed experimental data and conclusions and prepared the manuscript. All authors read and approved the final manuscript.
